# Case report: Paralysis after epidural analgesia due to a hemorrhage of pure epidural venous hemangioma

**DOI:** 10.3389/fneur.2022.1077272

**Published:** 2023-01-10

**Authors:** Jiahui Li, Pengyun Xie, Xiaolei Liu, Daheng Li, Jing Tang

**Affiliations:** Department of Anesthesiology, Affiliated Hospital of Guangdong Medical University, Zhanjiang, Guangdong, China

**Keywords:** extradural venous angiomas, epidural analgesia, myofascial pain syndrome, paralysis, pain

## Abstract

**Purpose:**

To report a case of sudden paralysis after epidural analgesia to raise awareness of the condition and the importance of early identification and appropriate treatment of extradural venous angiomas.

**Clinical features:**

A 28-year-old man with myofascial pain syndrome experienced paraplegia after receiving an epidural block for pain relief, which was later discovered to be caused by hemorrhage from extradural venous angiomas. Decompression surgery was performed immediately and successfully. A follow-up examination was performed 5 months after surgery. The patient reported improvement in urinary retention. The muscle strength in both his lower extremities had recovered to 4 out of 5 but still exhibited considerable residual spasticity.

**Conclusions:**

Before administering epidural analgesia to relieve undetermined pain, it is prudent to carefully weigh potential benefits against potential risks to patient health to minimize the likelihood of complications.

Spinal vascular malformations can be divided into four types: capillary, cavernous, arteriovenous, and venous. Extradural venous angiomas are a rare type of spinal vascular malformation but a serious cause of neurological deterioration, which includes sensory disturbance accompanied by radicular pain and/or paraparesis (1). In this study, we report on a spinal epidural venous angioma case in an adult who received epidural analgesia to relieve his progressive chest and abdominal pain but developed symptoms of bilateral rigidity and paralysis in the lower extremities after an operation. This case indicates that, for patients with chest and abdominal pain, we must immediately identify signs of rupture and bleeding of hemangiomas in the spinal canal and use epidural analgesia with caution.

## Case report

A 20-year-old man who had received epidural analgesia at the T6–7 position to relieve progressive chest and abdominal pain described the pain as a tense and discontinuous ache, especially when he laid down, and rated its severity as 9 out of 10. He had no history of using medications or having surgery and was not regularly taking any prescribed drugs. No deformity was found upon physical examination, but several tenderness points were found in the T3–T12 paraspinal muscles. The results of the chest and abdominal computed tomography (CT), routine blood studies, diastasimetry, four coagulation tests, and renal and liver function tests were normal. Myofascial pain syndrome was considered after the exclusion of organic disease. He was prescribed over-the-counter pain medication, but it did not relieve the pain. Based on previous treatment experience, trigger point injection is an effective therapeutic method for patients with mild myofascial syndrome, except for those suffering from acute pain. Considering the need for silver needle treatment under epidural anesthesia after the relevant examinations, the patient was administered epidural analgesia in advance to relieve the unbearable pain.

After assisting the patient in the knee-to-chest position, we monitored his vital signs through SpO2 and an electrocardiogram, and non-invasive blood pressure (NIBP) measurements were obtained every 5 min. After disinfection, a needle with 3 ml of 2% lidocaine was inserted. We performed puncture at the T6–T7 space *via* a median approach using an 18 G Tuohy epidural needle (TUORen Medical Instrument Group Co., Ltd., China). The loss-of-resistance to air technique was applied to confirm correct entry into the epidural space. A 4-cm epidural catheter was inserted into the back of the patient with the puncture needle facing the head and in a fixed position. After confirming the absence of the reflux of CSF and blood, we injected a test dose of 1% lidocaine (5 mL). An initial dose of 5 mL of lidocaine 1% and a continuous infusion of lidocaine 0.02% were programmed to be administered at a rate of 5 mL/h. After 15 min, the patient declared that the pain had been relieved. His vital signs were continuously monitored using SpO2 and an electrocardiogram and NIBP every 20 min after he returned to the ward. The sensation and motor function of his lower limbs were normal at the time.

**Figure 1:**
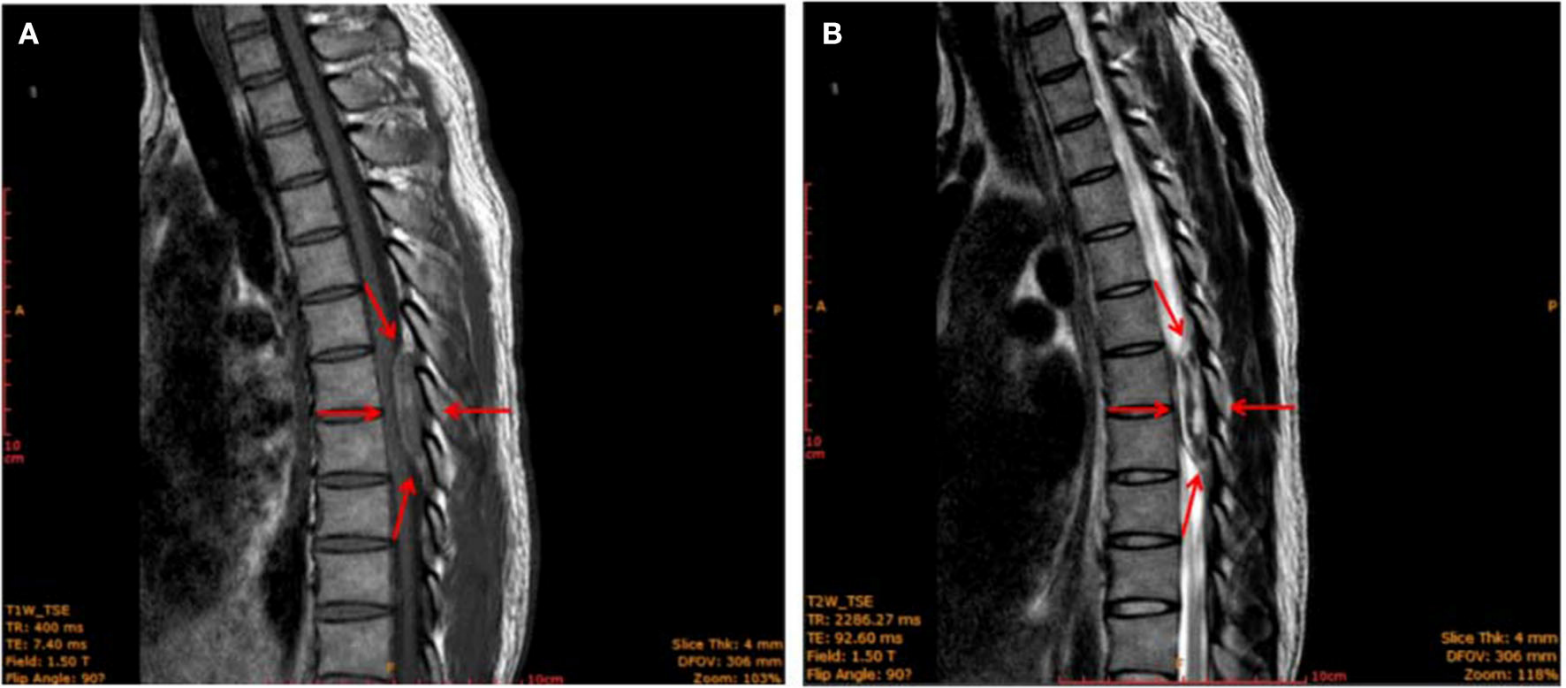
MRI image of the thoracic spine (sagittal plane) showing an epidural mass (red arrows) with a clear border measuring 11 × 16 × 11 mm located at the level of 2 thoracic vertebrae (T7–T8). The mass was iso-intense on T1-weighted images **(A)** and showed mixed-signal intensity on T2-weighted images **(B)**.

However, 4 h after epidural analgesia, the patient suddenly developed bilateral rigidity and paralysis in the lower extremities. A physical examination revealed numbness below the level of T6, grade I strength, and hyperactive deep tendon reflexes were noted in the bilateral lower extremities, while Babinski's response was also positive. The patient also reported difficulty with bowel or urinary retention or incontinence, and superficial sensations were abnormal in bilateral lower extremities. Gadolinium-enhanced magnetic resonance imaging (MRI) showed an epidural mass measuring 11 × 16 × 11 mm with a distinct border, which intensified at the T7–T8 extradural level. An iso-intense area was detected on the T1-weighted images (T1WI) and mixed-signal intensity on the T2-weighted images (T2WI). The T1-weighted transverse plane showed that the lesion occupied more than half of the T7–8 canal (Figure 1). Laminectomy, intravertebral mass removal, and spinal canal decompression were performed immediately and successfully. Multiple dilated, thin-walled vessels with hemorrhage and hematoma formation were observed through the histopathology of the extradural lesions. It also showed that CD31 was positively expressed in the epithelial cells of the cyst walls (Supplementary Figure 1). The extradural lesion was determined to be an extradural venous angioma. In patients with spinal epidural venous hemangiomas, a significant expansion of intralesional hemorrhage led to acute neurological deterioration. After 8 days of surgery, the sensory effect in both his lower extremities had recovered, and muscle strength was at 3 out of 5. Babinski's response was negative, and superficial sensations were normal in the bilateral lower extremities. However, no significant improvement in bowel and urinary retention was observed. A follow-up examination was performed 5 months after the surgery. The patient reported an improvement in urinary retention. Muscle strength in both his lower extremities had recovered to 4 out of 5, but the patitent still showed considerable residual spasticity.

## Discussion

Extradural venous angiomas are the most uncommon subtype of spinal vascular malformations (2, 3). Damage to the movement and differentiation of the primitive mesoderm is widely recognized as the cause of spinal vascular malformations. However, the etiology of spinal vascular malformations remains unclear (4). Only six cases of purely extradural spinal venous angiomas have been reported since 1978 during which the first case was reported by Decker et al. A review of these six cases showed that almost all patients presented with somatic pain, numbness, or limb weakness (2, 3). During the early stage, the patient's symptoms were fairly insidious, leading doctors to ignore them until the enlargement of the intralesional hemorrhage caused other symptoms (5). Spinal vascular malformations are difficult to diagnose preoperatively. During surgery, histopathology samples can be obtained to make a definitive diagnosis. Compressive epidural hemangiomas are best treated with surgical resection (4). The main differential diagnoses are epidural hematoma, spinal cord injury, neuroma, lymphoma, schwannomas, or meningioma. Compared with other common epidural spinal cord tumors, radiculopathy is the only clinical manifestation of epidural venous hemangiomas. Unfortunately, the MRI features of pure epidural venous hemangioma do not differ significantly from those of other space-occupying spinal cord lesions (6). Therefore, we may erroneously identify an epidural lesion as a spinal epidural hematoma preoperatively and thus fail to predict intraoperative bleeding in advance due to this misdiagnosis.

Myofascial pain syndrome lacks clear diagnostic criteria, and a characteristic symptom is a regional pain originating from a tender point located within the taut band of skeletal muscles (7). In this case, chest and abdominal CT, physical examination, and laboratory tests provide a normal result except for multiple tenderness points in the T3–T12 paravertebral muscles, which are classic symptoms of myofascial syndrome. On reflection, we aimed to determine whether radiculopathy and myofascial syndrome caused progressive xiphoid process pain and chest back pain in patients. The symptoms of radiculopathy, in this case, were related to chronic haemangioma, which is an atypical symptom and so insidious that the doctors ignored it.

Epidural analgesia has been shown to be a safe procedure that can produce equal or better pain relief than systemic opioids. However, some risks are still associated, including infection, intravascular or subdural medication injections, hematoma, air embolism, and direct nerve trauma. As epidural analgesia has become more standardized, major complications are now rare after surgery, especially the surgery that do not involve infection or bleeding resolve within 6 months (8, 9). According to statistical information available, the occurrence rate of spinal epidural hematoma after epidural anesthesia was 1/150,000 cases in patients who did not receive thromboprophylaxis (10). Some of the risk factors include coagulopathy, trauma, and vascular malformations (11, 12).

Our case is unique because the sudden paralysis after epidural analgesia is correlated with a purely extradural spinal venous angioma. This is the first report of a purely extradural spinal venous angioma with atypical symptoms that are difficult to distinguish from myofascial pain syndrome. Patients' perceptions of pain are highly subjective and have many functional implications and functional effects that cannot be detected through radiography. Radicular pain radiating from the trigger point is similar to myofascial pain syndrome. We should carefully perform differential diagnosis and conservative treatment until improvements are observed in relevant imaging examinations (13). To relieve undetermined pain, health physicians should weigh the potential benefits against potential risks to the patient's health to reduce the likelihood of complications before epidural analgesia.

## Data availability statement

The original contributions presented in the study are included in the article/Supplementary material, further inquiries can be directed to the corresponding authors.

## Ethics statement

The studies involving human participants were reviewed and approved by Institutional Review Ethics Committee of Guangdong Medical University. The patients/participants provided their written informed consent to participate in this study. Written informed consent was obtained from the individual(s) for the publication of any potentially identifiable images or data included in this article. Ethics approval number: YJLW2022005.

## Author contributions

JL and DL conceived the idea for this paper. XL and PX performed the literature search. JT and JL identified and/or managed the case. JL wrote the article. JT took responsibility for the veracity of the information mentioned in this report. All authors contributed to the article and approved the submitted version.
