# Supplementary material for: Case report: Paralysis after epidural analgesia due to a hemorrhage of pure epidural venous hemangioma
Source: Front Neurol. 2023 Jan 10;13:1077272. doi: 10.3389/fneur.2022.1077272 (PMC9871885; doi:10.3389/fneur.2022.1077272)
Supplement: Supplementary file 1 [file Image_1.pdf]

Sudden Paralysis After Epidural Anesthesia: A case report

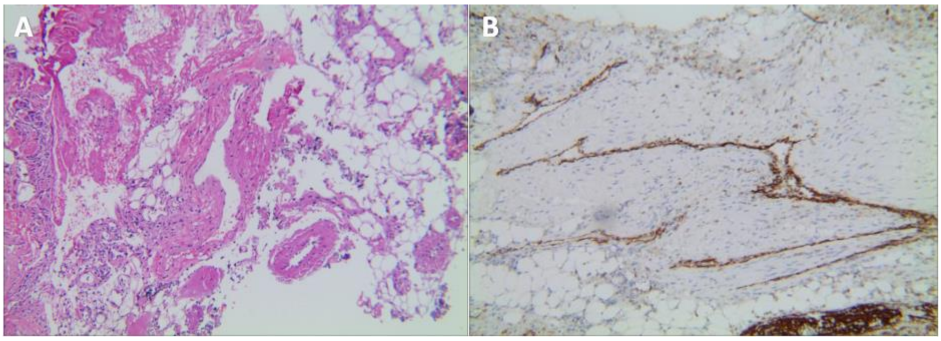

Supplementary Fig 1. A.Photomicrographs of the surgical specimens illustrating cystic dilated vessels with multiple layers of smooth muscle cells in the walls. H&E×100. B.The epithelial cells of cystic walls show positivity for CD31.CD31×100.
